# A65 HOSPITALIZATION RATES OF INFLAMMATORY BOWEL DISEASE DURING THE COVID-19 PANDEMIC: A POPULATION-BASED STUDY OF THE ORGANISATION FOR ECONOMIC CO-OPERATION AND DEVELOPMENT.

**DOI:** 10.1093/jcag/gwae059.065

**Published:** 2025-02-10

**Authors:** L M Wilson, J A King, E Buie, S Coward, A Shaheen, G G Kaplan

**Affiliations:** University of Calgary Cumming School of Medicine, Calgary, AB, Canada; Community Health Sciences, University of Calgary, Calgary, AB, Canada; Community Health Sciences, University of Calgary, Calgary, AB, Canada; Community Health Sciences, University of Calgary, Calgary, AB, Canada; Community Health Sciences, University of Calgary, Calgary, AB, Canada; Community Health Sciences, University of Calgary, Calgary, AB, Canada

## Abstract

**Background:**

Inflammatory bowel disease (IBD) places a significant burden on healthcare resources, requiring medical and surgical management in hospital. The COVID-19 pandemic further strained healthcare systems, but the impact of the global pandemic on IBD hospitalization rates remain unclear.

**Aims:**

To examine hospitalization rates for IBD prior to the COVID-19 pandemic (2017-2019) and during the pandemic (2020-2022) in Organisation for Economic Co-Operation and Development (OECD) countries.

**Methods:**

A population-based study was conducted using data from the 34 OECD member countries. Country-level data on annual IBD-related hospital discharge counts and rates (per 100,000 persons) were obtained. Mean hospitalization rates were calculated before (2017 to 2019) and during the pandemic (2020 to 2022). Incident rate ratios (IRR) comparing pandemic to pre-pandemic rates were calculated using Poisson regression, with 95% confidence intervals (CI). Countries without complete data during this the study period were excluded.

**Results:**

Overall, 22 countries were included, with 20 showing a statistically significant decrease in hospitalization rates during the pandemic. These decreases in hospitalization rates during the pandemic ranged from Lithuania’s IRR of 0.57 (95%CI: 0.53, 0.62) to Spain’s IRR of 0.94 (95%CI: 0.92, 0.95). Of the remaining two countries, Korea had an increase in hospitalization rates (IRR: 1.02; 95%CI: 1.00, 1.04; p=0.015), while Costa Rica showed no significant difference (IRR:0.93; 95% CI: 0.82, 1.04).

**Conclusions:**

IBD-related hospital discharge rates significantly decreased in most OECD countries during the pandemic. Future studies should explore whether the increased strain on healthcare systems during the pandemic shifted the management of IBD from hospitals to community-based care.

Table 1. IBD hospitalization discharge rates in OECD countries prior to (2017-2019) and during (2020-2022) the COVID-19 pandemic per 100,000 person-years.

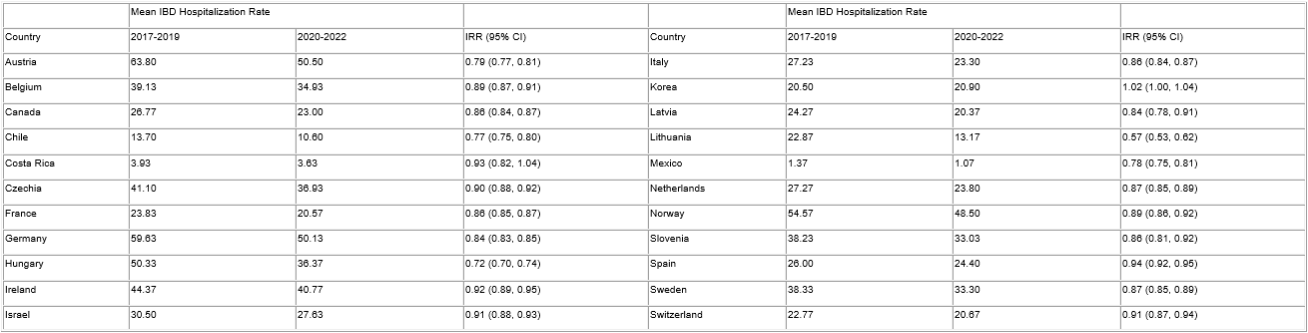

**Funding Agencies:**

None

